# A Review of Producer Adoption in the U.S. Beef Industry with Application to Enteric Methane Emission Mitigation Strategies

**DOI:** 10.3390/ani15020144

**Published:** 2025-01-09

**Authors:** Jaime R. Luke, Glynn T. Tonsor

**Affiliations:** 1 Department of Agricultural, Food, and Resource Economics, Michigan State University, East Lansing, MI 48824, USA; 2 Department of Agricultural Economics, Kansas State University, Manhattan, KS 66506, USA; gtonsor@ksu.edu

**Keywords:** cattle, climate, economics, greenhouse gas, incentives, synthesis, technology

## Abstract

Cattle are ruminant animals that produce enteric methane (CH_4_) emissions as a byproduct of their natural digestive process. U.S. beef producers have been receiving pressure to reduce production emissions. The scientific community continues to research and develop methods to reduce enteric methane emissions, but adoption of such strategies by U.S. beef producers remains unknown. We complete a review on producer adoption in the U.S. beef industry to shed light on potential factors that may impact the adoption of emissions-mitigating strategies by U.S. beef producers. After querying nine research databases, fifty-five studies were gathered and synthesized. These studies span the beef supply chain and focus on topics including feed additives, management practices, and reproductive technologies. Economic returns are a key driver of U.S. beef producer adoption decisions, with accompanying considerations for the impacts on consumer perceptions and demand. Segmentation in the U.S. beef supply chain, with animals typically changing ownership multiple times prior to slaughter, may result in challenges in tracing and verifying the adoption of climate-focused strategies. Targeting large-scale producers may be the most efficient avenue to achieving emissions reduction goals via the adoption of methane-mitigating strategies. Younger producers could additionally be a target demographic for adoption efforts.

## Simple Summary

Several strategies are being developed to reduce emissions from beef production. Future adoption of such strategies by U.S. beef producers is unknown. Many factors contribute to adoption decisions. This review synthesizes the recent adoption literature as it relates to the U.S. beef industry. Economic returns are a driving factor for U.S. beef producers to adopt new practices and technologies. Adoption also hinges on the quality and availability of information. The highly segmented nature of the U.S. beef supply chain may hinder the widespread adoption of climate-focused strategies. Operation size is a key characteristic influencing adoption decisions, with larger operations typically being more willing to adopt new practices and technologies. In addition, younger producers are oftentimes more likely to adopt these practices than their more senior counterparts. Numerous other factors, spanning geographic location to educational attainment, additionally impact producer adoption decisions. Limited research has been performed addressing the adoption of emissions-reducing strategies in U.S. beef production. Going forward, bolstering research in this area could provide vital information for policymakers, beef industry participants, and the scientific community.

## 1. Introduction

Global demand for meat is projected to increase 57% by 2050 compared to 2005 levels [1]. Consequently, the production of beef has been rapidly increasing, with global beef production more than doubling from 1960 to 2010 [2]. Beef production is economically important to agricultural economies around the world, especially the United States as the world’s leading beef producer [3]. The U.S. beef industry has faced numerous challenges over the past decades spanning from natural disasters (e.g., droughts and extreme heat) to diseases (e.g., bovine respiratory disease (BRD)). Most recently, U.S. beef producers have been receiving calls to change practices in the wake of climate change concerns.

**Figure 1:**
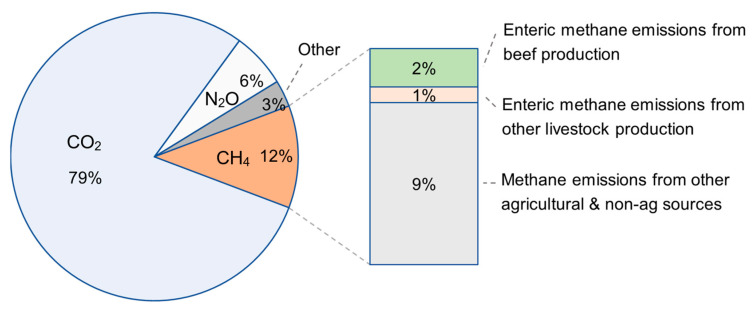
Total U.S. greenhouse gas (GHG) emissions. (Percentages represent total GHG emissions, not CO_2_ equivalents).

Beef cattle are ruminant animals that emit enteric methane (CH_4_) emissions as a natural byproduct of their feed digestive process. More specifically, food consumed by cattle is decomposed and fermented by microbes in the digestive tract and rumen. This process produces energy and nutrients for the animal, but methane is additionally created and released from digestible energy loss [4]. Methane is a greenhouse gas (GHG), meaning it traps heat in the Earth’s atmosphere and contributes to the warming of the planet. Of total U.S. GHG emissions, roughly 12% come in the form of methane, second only to carbon dioxide (CO_2_) at 79%. Breaking that figure down further, approximately 2% of U.S. GHG emissions can be attributed to enteric methane emissions from beef production [5], as illustrated in Figure 1. Methane is a highly potent GHG, with a warming potential approximately 27 to 30 times that of carbon dioxide [6]. This makes it a target for many GHG emissions reduction campaigns, both in the United States and around the globe.

Given the negative environmental impact of methane and other GHG emissions, many entities have created goals to reduce emissions. The Global Methane Pledge, aiming to reduce global methane emissions by 30% by 2030 as compared to 2020 levels, has been signed by the United States and over 150 other countries [7]. Additionally, over 1500 private sector firms have pledged net zero GHG emissions by 2050 [8]. U.S. policymakers have proposed the Enteric Methane Innovation Tools for Lower Emissions and Sustainable Stock (EMIT LESS) Act [9,10]. If enacted, the EMIT LESS Act would provide funding to expand research for emissions-reducing feed additives and create voluntary incentives for beef producers to adopt emissions-reducing strategies.

The scientific community continues to devote resources to researching and developing strategies that aim to reduce enteric methane emissions [11,12]. Among these are the use of synthetic feed additives, such as 3-Nitrooxypropanol (3-NOP) [13]. While not commercially approved for use in U.S. beef production, this additive has been approved in other major beef producing countries, including Australia, Brazil, and the European Union [14]. Natural feed additives are also being investigated, including red seaweed [15] and garlic and citrus [16]. Genetic selection has additionally been explored [17,18] as well as diet reformulation [19]. Other regenerative livestock production practices, including grazing management strategies, are likewise being researched to mitigate emissions [20].

A necessary condition for any of these strategies to be adopted is that they be scientifically sound. However, this condition alone is not sufficient. They must also be economically viable to be widely adopted by U.S. beef producers. As stated previously, enteric methane emissions are, in essence, an energy loss for ruminants. This leads to inefficiency in production that is detrimental to producer profitability. Curbing emissions via methane mitigation strategies could potentially decrease inefficiency and improve productivity. This could present a dual benefit (i.e., a “win-win” benefitting producers economically and the environment via reduced emissions). However, strategies must exhibit such results to be economically viable for producers. Examples exist of scientific discoveries that were never adopted in practice because they ultimately did not make “economic sense” to practitioners [21]. Thus, while many potential strategies exist that could curb the environmental impact of beef production, the question remains: will U.S. beef producers adopt any of the proposed strategies? 

Currently, scant research has been undertaken on the adoption of emissions-reducing strategies by U.S. beef producers. Our review found few studies in this space, but we acknowledge that other beef producer adoption pieces are available in the literature. Therefore, the objective of this work is to complete a review of producer adoption in the U.S. beef industry to shed light on potential factors that may impact the adoption of emissions-reducing strategies by U.S. beef producers going forward. With these findings, we aim to identify potential target producer segments that could be most willing to adopt new practices and technologies. Additionally, we illuminate key areas for future research to galvanize efforts to fill knowledge gaps present in this space.

The remainder of the paper is presented as follows: firstly, procedures to narrow research selection are outlined, followed by a synthesis of identified record findings. We then summarize literary works into relevant categories. Results of the review are presented, followed by a discussion of “target” adopters. Finally, recommendations are made for future research, and conclusions are drawn.

## 2. Materials and Methods

### 2.1. Selection of Research

An online database search was completed using the following commonly consulted economic-specific and noneconomic-specific literature searching platforms: AgEconSearch, Agricola, CAB Abstracts, Cambridge, EconLit, JSTOR, PubMed, Scopus, and Wiley. Records were filtered in August 2024 using the following text string (beef AND adopt* AND econ* AND United States). We limited searches to records written in English and free to access. For academic journal articles, this includes only publications available via open access. Additionally, the timespan January 2010 to August 2024 was enforced. For CAB Abstracts, Cambridge, JSTOR, Scopus, and Wiley, searches were further limited to those that included (beef) in the title. For the Scopus search, limits were additionally imposed to include (adopt*) in the title, abstract, or keywords. These additional limitations were introduced given the immense number of unrelated studies retrieved when not in place. Additional modifications were not made to the remaining search platforms (i.e., AgEconSearch, Agricola, EconLit, and PubMed).

**Figure 2:**
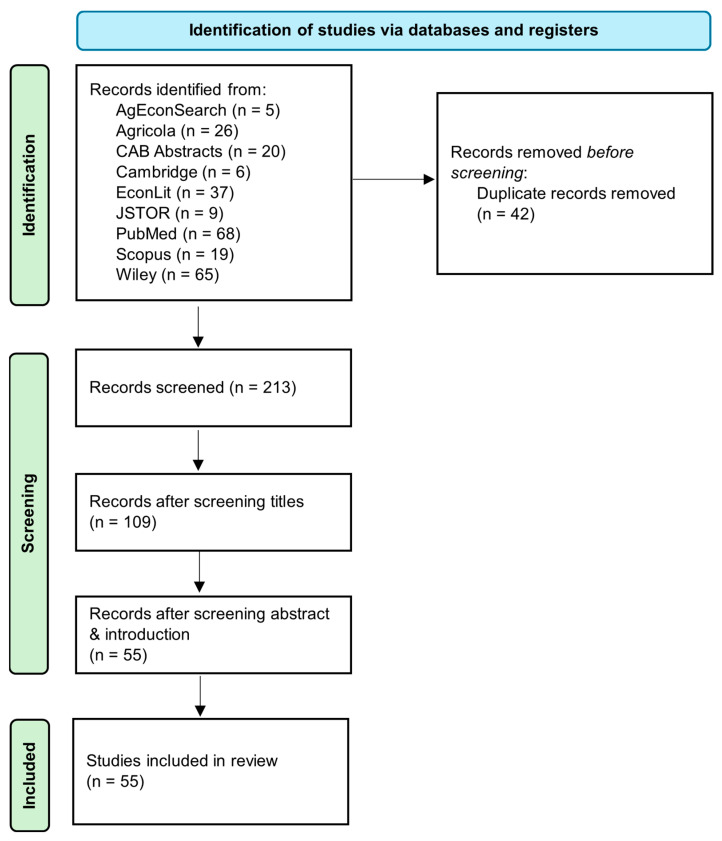
Identification, screening, and inclusion of studies used in synthesis review.

A total of 255 records were retrieved following these filtering procedures. We first removed 42 duplicate records so that 213 remained. The titles of each of these remaining records were scanned for relevance to this study. Those that were deemed irrelevant were further purged. Many of the records that were dropped at this stage were examining topics outside of the United States (e.g., “Farm and Socio-Economic Characteristics of Smallholder Milk Producers and Their Influence on Technology Adoption in Central Mexico”) or successfully navigated filtering because the word beef was used as a phrasal verb (e.g., “Does network closure beef up firms’ performance?”). Others were obviously outside the scope of this research (e.g., “Cardiovascular health and cancer risk associated with plant-based diets: An umbrella review”). Upon completion of title screening, 109 records remained. Abstracts and introductions of the remaining records were read and checked for relevance. Here, relevant works were required to address either “who” adopts a practice or technology in U.S. beef production or “why” a practice or technology is adopted in U.S. beef production. Some form of economic reference was required for inclusion in the final review, albeit broadly defined. Studies spanning from producer surveys, to those highlighting economic feasibility or cost-benefit analyses, to complex econometric models were deemed acceptable. After this screening process, 55 records remained. A flowchart summarizing identification, screening, and inclusion is presented in Figure 2.

### 2.2. Synthesis of Research

In synthesizing the studies, every attempt was made to forgo any a priori assumptions to maintain the integrity of our results. The 55 records used in this synthesis review are varied in nature. Some came from peer-reviewed outlets while others did not. While all focus on U.S. adoption, several are specific to certain states or regions. We also found a mix of publications from journals in multiple disciplines. The decomposition of sources into relevant categories is shown in Table 1, with further discussion of these categories following.

#### 2.2.1. Segments of U.S. Beef Supply Chain

The U.S. beef supply chain is highly segmented. Seedstock operations produce bulls or semen used to service cows on cow–calf operations. Commercial cow–calf operations typically produce weaned calves. These calves can be sold to stocker or backgrounding operations or placed directly in feedlots. Stockers and backgrounders rely on forages for feeding the weaned calves but additionally supplement protein, energy, vitamins, and minerals as needed. The feedlot stage of production usually spans the last few months before slaughter and is a period of efficiently adding weight to the animal’s carcass [22]. Variations of this supply chain exist for other niche sectors of U.S. beef production (e.g., grass-fed beef), but the primary production of U.S. beef follows the path described here. 

Importantly, each segment of the U.S. beef supply chain has unique characteristics that ultimately impact the ability of a producer to adopt or not adopt a practice. Most of the research explored in this synthesis review focuses on either cow–calf production (23), is not specific to one segment of the supply chain, or includes multiple segments (28). A few studies are specific to feedlot production (4), while no studies directly address adoption specific to stocker or backgrounding operations alone.

#### 2.2.2. Peer-Reviewed Versus Not Peer-Reviewed

The majority of studies used in this review are peer-reviewed (47). Often, these works include robust analyses with scientific audiences in mind. They frequently employ highly technical language and advanced methodologies unknown to the untrained reader. The remaining 8 studies in this review are not peer-reviewed. Typically, these publications are magazine articles or short summaries of more extensive research papers. They usually are jargon-free and understandable to the general public.

#### 2.2.3. Contribution of Research

As previously noted, only studies that explain “who” adopts a practice or technology in U.S. beef production or “why” a practice or technology is adopted in U.S. beef production were included in this review. Some studies addressed both but were classified by their primary “who” or “why” research output.

#### 2.2.4. Census Region

**Figure 3:**
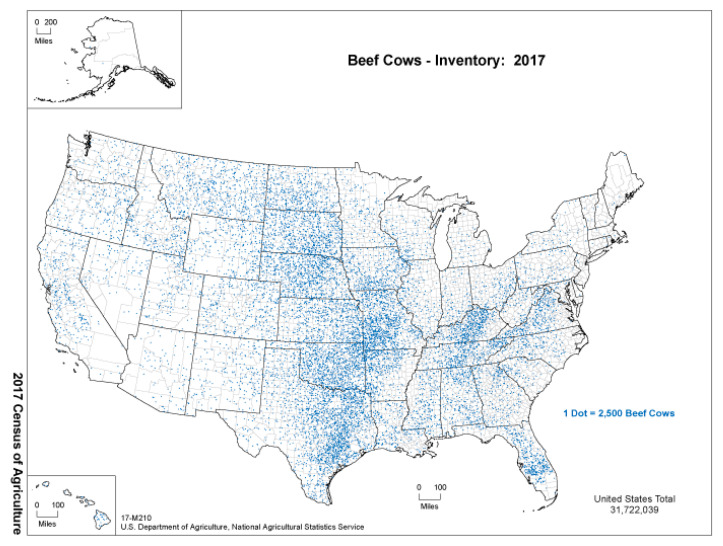
U.S. beef cow inventory according to the 2017 USDA Census of Agriculture (Source: USDA National Agricultural Statistics Service).

Beef production is possibly the most geographically widespread agricultural production sector in the United States. Figure 3 graphs the 2017 beef cow inventory. As shown by the figure, every U.S. state is home to beef cows, with the greatest number of beef cattle concentrated in the Midwest and Plains region (i.e., Texas, Oklahoma, Missouri, etc.). Even so, pockets of dense beef cow populations exist elsewhere, including parts of central Florida and Kentucky. As calves progress through the beef supply chain, they become more concentrated to the middle of the U.S. where the majority of feedlots and beef processors are located.

As such, many studies used in this review span various geographies, reflecting the widespread nature of U.S. beef production. The geographic region with the greatest number of studies is the South (7), which as shown in Figure 3 is home to a large number of the nation’s beef cattle population. Other studies are more general and focus on the entire country rather than narrowing the focus to a specific geography.

#### 2.2.5. Practice or Technology

Beef production is a complex process with many potential practices or technologies that can be employed by producers. We narrow the topics covered in the studies included in this review to a few overarching classifications. Feed additives include products like the previously discussed 3-NOP or beta-agonists (growth-promotants) like Zilmax^®^. Management practices could include, for example, dehorning, deworming, or castration. This classification could also include administering vaccines, such as an *E. coli* vaccination. The reproduction and genetics classification spans from artificial insemination (AI) to genomics testing. Traceability and precision livestock farming (PLF) include blockchain technology and virtual fencing, among others. Some studies cover multiple classifications or are not a seamless fit for any of the classifications (e.g., Beef Quality Assurance (BQA) certification). These were assigned to the multiple/other classification.

#### 2.2.6. Publication Outlet

Most of the studies used in this synthesis review are published via academic journals. Of the total, 19 originate from journals in agricultural economics, 26 come from animal science, and the remaining works are published in journals or other outlets outside either of the aforementioned disciplines (e.g., Smart Agricultural Technology). The blend of disciplines provides insights from numerous economists and animal scientists on a topic that is of keen interest to both parties.

#### 2.2.7. Year Published

We found that over half of the studies in our selection were published in the past five years (28) with the remaining 27 being published in the decade prior (2010–2019). Given the dynamic nature of U.S. beef production, having more recent studies included in the set is likely a positive as the industry is ever evolving. 

## 3. Results and Discussion

Initially, we explore why U.S. beef producers elect to adopt practices or technologies. This examination is followed by a summary of insights on the common characteristics of who adopts practices or technologies in U.S. beef production. We conclude the results section with a discussion for pertinent future research in this space.

### 3.1. Why U.S. Beef Producers Adopt Practices or Technologies

#### 3.1.1. Economic Returns

Economic returns are a driving factor for U.S. beef producers to adopt a new practice or technology [23]. Net profit is calculated by subtracting costs from revenues. Hence, strategies that decrease the cost of production or increase revenues are of interest to producers seeking to maximize profits. 

Consider first the cost portion of the net profit equation. Major costs to U.S. beef producers are feed, labor, and land, among others. Certain reproductive technologies decrease costs and spur producer adoption simply from a cost-saving standpoint [24,25]. However, many new practices or technologies have the opposite impact. That is, producers are hesitant to adopt given the upfront costs. Examples of this span from precision livestock farming tools [26], to genomic testing [27], to artificial insemination [28], and to emissions reduction strategies [29]. 

Yet, increased costs can occasionally be offset by even larger increased revenues, resulting in economically sound adoption decisions. Revenue generated along the beef supply chain is influenced by a number of factors. Perhaps the most important is consumer demand. Consumers influence beef production practices by paying more for beef products with various attributes. Niche markets provide opportunities for producers to differentiate their end-product to garner premiums in the marketplace [30]. For example, beef produced with climate-based production practices, such as feeding them 3-NOP, could be marketed with climate labels at a premium to climate-conscious consumers. Niche markets require greater coordination and often more labor to process and market the differentiated products. This remains a drawback for niche market sales for cow–calf producers, as they do not produce an “end product” and typically market their calves through traditional market channels that forgo the opportunity for premium-based “attribute” sales [31]. 

More generally, consumer perceptions play a role in producer adoption decisions. Implementing animal identification technology could increase consumer confidence in the U.S. meat system [32]. Conversely, potential negative consumer perceptions to new technologies can also create apprehension when considering adoption decisions [33]. International trade considerations are also of interest to U.S. beef producers. Market access, both domestically and internationally, increasingly impacts producer profitability. If other countries implement practices and technologies that become “industry standard”, the United States could lose market share if those standards are not likewise adopted in domestic production [34,35]. Moreover, adopting strategies that increase the price of U.S. beef also risks decreasing the competitiveness of U.S. beef on the global market, which again is detrimental to the producer’s profitability potential [36]. 

Economic returns may be influenced by producers adopting a combination of practices and technologies (e.g., improving genetic selection and management practices). Past work has found that the independence of beef producer adoption decisions is too simple of an assumption [37]. Rather, complex adoption possibilities exist [38,39,40], with the potential to improve the productivity and the sustainability of U.S. beef production simultaneously. 

Other alternatives exist to spur adoption. While beef producers show preference for value created via marketplace premiums, a subset of producers also responds to economic incentives in the form of government support which is used to spur adoption [41]. Further, mandating producers to adopt certain practices generates the adoption outcome but would also increase the price of beef assuming producers are already producing at the highest efficiency level. Given the complexity of the beef supply chain, mandating a specific sector or a specific practice could also create structural changes in the beef market [42]. 

#### 3.1.2. Information

Both the quality and the availability of information impact beef producer adoption decisions, as simply lacking awareness of an opportunity can impede adoption [43]. Producers seek information from various sources and have expressed apprehension about the learning curve associated with new technologies [26]. Veterinarians often serve as key advisors for producers [44]. In addition, university extension personnel also play an important role in providing unbiased information and often serve as a connection between research occurring at U.S. universities and those active in production agriculture [45,46]. 

Information quality depends partially on the quality of questions being asked. Research on lesser-known products or technologies for adoption could ultimately influence the U.S. beef production landscape [47], but both time and financial constraints exist. As such, there are limitations in the breadth of information generated, which increases the need for research that joins together multiple disciplines as well as practitioners in the research process [48]. Public–private partnerships also play an important role [49], and studies at the national and sub-national level can provide an enhanced value [50]. In essence, the U.S. beef production system is extremely complex, which underscores the need for coordination in research, development, and outreach efforts.

#### 3.1.3. Segmentation of the Beef Supply Chain

The existing literature has highlighted that beef producers often lag dairy producers in adopting practices and technologies [51,52,53]. This finding may be partially due to the segmented nature of the beef supply chain as alluded to earlier. Prior to slaughter, cattle may change ownership numerous times as they move from cow–calf operations to stocker or backgrounding operations and finally into feedlots. This segmentation creates difficulties for coordinating practices, as production decisions made early in the production process can have impacts down the supply chain. Conversely, adoption of practices post-harvest can impact consumer demand and in turn the demand for cattle earlier in the supply chain [33,54,55]. The traceability and verification of adoption decisions, though costly, are integral if economic returns are created as a result.

Oftentimes, the impacts of producer adoption decisions on downstream agents (e.g., grocery retailers) are different from those on upstream agents (e.g., feedlots) [56]. Quantifying the full market impacts of such adoption decisions can be cumbersome. However, previous economic works, typically employing equilibrium displacement models (EDMs), have done so [32,34,57]. For adoption to occur at the beginning of the supply chain, the value of adoption must reach the adopting agent. Hence, sharing generated value with upstream agents is paramount for successful adoption [58].

### 3.2. Who Adopts Practices or Technologies in U.S. Beef Production

Several common characteristics exist among those who are keen to adopt new practices and technologies and those who are not. We begin our discussion by addressing operation size. The literature suggests that larger producers are more likely to adopt new practices and technologies in beef production than smaller producers [41,43,59,60,61,62,63,64]. This could potentially be attributed to the implementation costs. Depending on the practice or technology, if a producer has a greater number of head to implement a practice or a technology on, the implementation cost per head could be much lower for a large producer [65]. Risk tolerance could also play a role. Large producers are more likely to be engaged in risk-mitigating behaviors for their operation [66], so they may be more willing to take on risk associated with a new practice or technology adoption. 

We find that younger producers are often more willing to adopt new practices or technologies [67,68,69,70], while tradition (i.e., “doing things how we’ve always done them”) does seem to be part of the adoption story on some operations as well [71]. Studies have additionally shown that producers who attend extension programing and are more highly educated and/or informed are more likely to adopt new practices or technologies in beef production [72,73,74,75]. Geographic location in the United States can also be a predictor of adoption outcomes, with producers in the Midwest and West more likely to adopt technology, management practices, and production systems compared to those in the Southeast [61]. This could potentially result from climate differences in some instances, but further research is needed to better understand this finding. Cow–calf producers (as opposed to those further down the supply chain) are more likely to keep individual animal records [63], which could improve their ability to engage in tracing and identifying specific practices used in production. Stockers, conversely, are more likely to adopt implants or ionophores than any other segment of the supply chain [63], likely because of the focus on adding weight to the carcass in this production stage. Grass-fed beef producers are more likely to adopt advanced reproductive management practices [76], but this production approach represents only a fraction of U.S. beef producers. Lastly, as a producer’s share of income coming from beef production increases, the more likely they are to be adopters [43,73]. Potentially, those who depend more heavily on beef production returns invest more focus and time in management and production decisions.

This exploration of producer characteristics sheds light on the potential “early adopters” of climate-focused practices or technologies. Targeting large-scale producers may be the most efficient avenue to achieving emissions reduction goals, as large producers are creating more emissions via having a greater number of cattle, and they are additionally willing to adopt strategies at a lower incentive [41]. Younger producers could also be a key target demographic. Although they may manage smaller operations, this group may be more open to the prospect of new beef production practices and technologies. Importantly, circling back to the earlier point, incentives must align for adoption to occur regardless of beef producer characteristics.

### 3.3. Knowledge Gaps and Necessary Future Research 

As previously stated, little research exists that examines the economic component of livestock emissions and associated producer adoption of introduced enteric methane emissions-reducing strategies. Thus, we suggest bolstering research in this area. Potential future work could investigate the adoption of strategies across the beef supply chain, as notable differences exist among the segments. Additionally, work that seeks to understand differences in the potential producer adoption of various products and practices could be of value. That is, would a feedlot producer rather feed 3-NOP or seaweed? And at what incentive level? Legislation like the EMIT LESS Act may provide funds for researching the use of emissions-reducing feed additives, but we reiterate that the relevance of such feed additives hinges on their economic viability. There is an opportunity for studying beef producer willingness to engage in carbon credit trading and supply chain insets. Could commoditizing carbon produce a sustainable stream of additional revenue for beef producers? The U.S. public’s willingness to support producers, whether via retail premiums or via government-based provisions (e.g., subsidies), presents yet another potential research project in this space. This list is not exhaustive as this topic is ripe for the investment of time and resources. 

## 4. Conclusions

The U.S. beef industry faces pressure from outside entities to reduce enteric methane emissions. Numerous strategies are being researched and developed to reduce said emissions, but the economic incentives necessary to spur widespread adoption are currently lacking. Economic returns are a key driver to producer adoption of new practices and technologies. Strategies that reduce costs or increase revenues are the most likely to be implemented as they make “economic sense”. Economic returns are influenced by many factors, including consumer perception and demand and international market access. The quality and availability of information also have impacts on producer adoption decisions, with extension personnel and other producer “advisors” (e.g., veterinarians) being identified as trusted sources of information in the adoption decision-making process. We further find that the segmentation of the beef supply chain hinders the adoption of some practices and technologies because traceability and verification can be especially burdensome and costly. U.S. beef producers most likely to adopt new practices and technologies are typically those who have large operations or are younger. Potentially, large operations (i.e., those with more head of cattle) could be a sensible target for adopting emissions-reducing strategies because they emit more methane than producers with small operations and may be willing to adopt strategies at a lower incentive level. A number of other producer characteristics are likewise pertinent in this discussion, ranging from education level to geographic location. 

Research questions abound concerning producer adoption of methane mitigating practices and technologies. Our hope is that this study will provoke future work that addresses key questions present in this area.

## Figures and Tables

**Table 1:** Decomposition of sources used in the synthesis review into the relevant categories and classifications.

Classification	Cow–Calf	Feedlot	Not Segment-Specific/ Multiple	Total
*Peer-Review Status*				
Yes	18	4	24	47
No	5	0	3	8
*Contribution*				
“Who” Adopts	12	1	7	20
“Why” Adoption Occurs	11	3	20	35
*U.S. Census Region*				
Midwest	3	0	0	3
Northeast	0	0	0	0
South	7	0	0	7
West	2	0	1	3
General U.S.	11	4	26	42
*Practice or technology*				
Feed Additive	0	2	2	4
Management Practices	8	2	5	15
Reproduction/Genetics	5	0	6	12
Traceability/PLF	1	0	6	7
Other/Multiple	9	0	8	17
*Publication Outlet*				
Agricultural Economics	7	2	9	19
Animal Science	14	0	12	26
Other	2	2	6	10
*Year Published*				
2010–2014	4	1	8	13
2015–2019	9	1	3	14
2020–2024	10	2	16	28

Note: Numbers in columns are the count of studies in each category or classification. Numbers sum to 55 for each category. PLF refers to precision livestock farming. No studies were specific to the stocker/backgrounding segment of production, so this category was excluded from the table.

## Data Availability

No new data were created or analyzed in this study. Data sharing is not applicable to this article.
